# A spectroscopic theory for how mean rainfall changes with surface temperature

**DOI:** 10.1126/sciadv.adv6191

**Published:** 2025-05-09

**Authors:** Sean Cohen, Robert Pincus

**Affiliations:** Lamont-Doherty Earth Observatory, Columbia University, Palisades, NY 10964, USA.

## Abstract

Surface warming is projected to increase global mean rainfall primarily by increasing the radiative cooling of the atmosphere. However, the radiative mechanisms which cause cooling to increase are not well understood. Here, we show that changes in cooling are driven primarily by changes in atmospheric opacity, particularly within the water vapor window. This suggests that changes in mean rainfall are primarily controlled by the thermodynamic and spectroscopic properties of Earth’s main greenhouse gases: water vapor and carbon dioxide. Consistent with comprehensive general circulation models, our results explain why mean rainfall increases with surface warming at about 2% per kelvin, why this rate is largely unchanged over numerous doublings of atmospheric carbon dioxide, and why mean rainfall decreases in hothouse climates.

## Teaser

The spectroscopic and thermodynamic properties of Earth’s atmosphere explain why rainfall increases with surface temperature.

## INTRODUCTION

Globally integrated rainfall is expected to increase as the climate warms and surface temperatures rise. This response has been evident in simulations since the first attempts to understand climate change (*1*, *2*) and holds across a broad hierarchy of models. The rate at which mean rainfall changes with surface temperature, termed hydrological sensitivity, is robustly estimated at ~2%/K (*3*–*5*).

Precipitation and its rate of change with surface temperature are constrained by the atmosphere’s energy budget. Conservation of energy within the atmosphere requires that surface sensible heating (direct warming of the air by the surface) and latent heating (evaporation of water at the surface which subsequently falls as precipitation) be balanced by column-integrated radiative cooling (*6*–*8*). At surface temperatures near that of Earth’s current climate, sensible heating is small relative to latent heating (*9*). Mean precipitation is thus approximately equal to column-integrated radiative cooling, and, to the extent that changes in sensible heating with surface temperature are also much smaller than changes in latent heating, increased precipitation is a consequence of increased radiative cooling. Mean rainfall is also affected by changes in atmospheric composition, which, independent of any change in surface temperature, affect the atmosphere’s energy balance (*10*). A similar energetic constraint can be leveraged using the surface energy budget (*11*, *12*), although the surface takes longer to equilibrate than the atmosphere and its energetic budget is more heavily affected by clouds (*13*).

Recent work (*8*) proposes an explanation as to why column-integrated radiative cooling increases with surface temperature at roughly 2%/K. Because water vapor concentration is closely tied to temperature, radiative cooling profiles within the troposphere also depend primarily on local temperature (*14*, *15*). Cooling vanishes at a roughly fixed temperature at the top of the troposphere (*16*), so column-integrated cooling depends only on how quickly the cooling profile increases with temperature near the surface. Calculations show this growth to be roughly quadratic, which, combined with the roughly 100-K range of temperatures spanned by the troposphere, yields a hydrological sensitivity of about 2%/K (*8*). This view, while fruitful, explains one result emerging from simulations—that precipitation increases at about 2%/K—with another—that cooling increases roughly quadratically with temperature.

Here, we show that hydrological sensitivity is primarily controlled by the thermodynamic and spectroscopic properties of Earth’s main greenhouse gases: water vapor and carbon dioxide. Idealized models of radiative transfer, spectroscopy, and atmospheric structure suggest that hydrological sensitivity is a consequence of changes in atmospheric opacity, particularly within the water vapor window in the infrared. We qualitatively describe the spectral mechanisms which control hydrological sensitivity and derive what they imply quantitatively about its magnitude and surface temperature dependence. These processes explain, from first principles, why mean rainfall grows at a rate of about 2%/K, why this rate is largely unchanged over numerous doublings of atmospheric carbon dioxide (*17*), and why hydrological sensitivity reverses sign in hothouse climates (*18*).

## RESULTS

### Changes in transmission control changes in mean rainfall

When a forcing agent such as carbon dioxide induces a surface temperature change $\Delta T_{s}$, the change in net (shortwave plus longwave) clear-sky column-integrated radiative cooling ΔQ is given by the sum of the temperature-driven response $\frac{dQ}{{\mathrm{dT}}_{s}}\Delta T_{s}$ (an analog of climate feedback) and the direct response $\Delta Q_{\text{direct}}$ (an analog of radiative forcing, i.e., the change in cooling independent of any change in surface temperature) (*19*). In the absence of clouds and surface sensible heating (assumptions that we relax below), ΔQ yields an equivalent change in mean rainfall ΔP

$$L\Delta P\approx \Delta Q=\frac{dQ}{{\mathrm{dT}}_{s}}\Delta T_{s}+\Delta Q_{\text{direct}}$$ (1)

where L is the latent heat of vaporization. Putting the direct response aside for the moment, $\frac{dQ}{{\mathrm{dT}}_{s}}$ can be decomposed spectrally by considering the contribution to shortwave heating ($H_{\text{sw}}$) and longwave cooling ($Q_{\text{lw}}$) from each wave number (inverse wavelength) ν. In the shortwave, heating is dominated by absorption by water vapor in the near-infrared. Neglecting surface reflection and atmospheric scattering, the spectrally resolved, column-integrated sensitivity of clear-sky shortwave heating to changes in surface temperature is

$$\frac{dH_{\nu ,\text{sw}}}{{\mathrm{dT}}_{s}}=-I_{\nu }\frac{dT_{\nu }}{{\mathrm{dT}}_{s}}$$ (2)

where $I_{\nu }$ is the spectrally resolved incident solar flux and $\frac{dT_{\nu }}{{\mathrm{dT}}_{s}}$ is the change in atmospheric transmission with surface temperature (*20*).

The longwave contribution to $\frac{dQ}{{\mathrm{dT}}_{s}}$ can be expressed analogously by making two key simplifications. One is that radiative cooling is controlled by the cooling of the atmosphere to space (*21*) (this assumption is assessed in the Supplementary Text), which allows us to express the column-integrated longwave cooling as

$$Q_{\nu ,\text{lw}}=\int_{T_{\nu }}^{1}\pi B_{\nu }\left[T\left(T\right)\right]dT$$ (3)

where $B_{\nu }$ is the Planck function and T(T) is the temperature at transmission level T. The second is that atmospheric opacity is dominated by water vapor. Being condensable at earth-like conditions, water vapor’s concentration in earth’s atmosphere is closely tied to temperature. Neglecting any pressure dependence of absorption and assuming no change in relative humidity with surface temperature (*22*), this tight coupling implies that the contribution to atmospheric cooling from a given transmission level does not change with surface temperature {$\frac{{\mathrm{dB}}_{\nu }\left[T\left(T\right)\right]}{{\mathrm{dT}}_{s}}=0$} (*8*, *14*, *15*). With these simplifications, spectrally resolved, column-integrated longwave cooling sensitivity—that is, the derivative of Eq. 3 with respect to surface temperature—is well approximated by

$$\frac{dQ_{\nu ,\text{lw}}}{{\mathrm{dT}}_{s}}=-\pi B_{\nu }\left(T_{s}\right)\frac{dT_{\nu }}{{\mathrm{dT}}_{s}}=\pi B_{\nu }\left(T_{s}\right)\frac{d\epsilon_{\nu }}{{\mathrm{dT}}_{s}}$$ (4)

where $\epsilon_{\nu }=1-T_{\nu }$ is the atmospheric emissivity at wave number ν (see Materials and Methods).

Equations 2 and 4 illustrate that, to first order, the radiative constraint on hydrological sensitivity depends only on the change in column transmission with surface temperature weighted by a radiative source (sunlight or surface Planck emission) (*8*, *23*). Surface warming increases rainfall by decreasing atmospheric transmission in spectral regions with substantial surface Planck emission and, simultaneously, mutes this response by decreasing atmospheric transmission in spectral regions with substantial incoming sunlight.

### Qualitative understanding: Connection to the closing of spectral windows

What sets the location and extent of the spectral regions that drive hydrological sensitivity? Atmospheric transmission decreases most with surface warming in spectral regions transitioning from optically thin (i.e., window regions) to optically thick (i.e., non-window regions) (*23*). Idealized models of spectroscopy and atmospheric structure, described qualitatively in this section and quantitatively in the next, suggest that the material properties of water vapor and carbon dioxide control how these spectral windows close and, thus, how rainfall changes with surface temperature.

In the longwave, the fact that water vapor’s contribution to the column optical depth ($\tau_{\nu ,\text{wv}}$) strongly depends on surface temperature, while that of other, non-condensable species ($\tau_{\nu ,o}$) does not, leads to a more explicit form of Eq. 4

$$\frac{dQ_{\nu ,\text{lw}}}{{\mathrm{dT}}_{s}}=\underset{\text{thermodynamic}}{\underbrace{\frac{d\text{ln}\mathrm{WVP}}{{\mathrm{dT}}_{s}}}}\underset{\text{spectral}}{\underbrace{\pi B_{\nu }\left(T_{s}\right)\overset{{\text{CO}}_{2}}{\overbrace{e^{-\tau_{\nu ,o}}}}\overset{H_{2}O}{\overbrace{\tau_{\nu ,\text{wv}}e^{-\tau_{\nu ,\text{wv}}}}}}}$$ (5)

Here, we have substituted $e^{-\left(\tau_{\nu ,\text{wv}}+\tau_{\nu ,o}\right)}$ for $T_{\nu }$ and assumed that the optical depth of water vapor is the product of the column water vapor path (WVP) and an absorption coefficient depending only on wave number (this neglects the temperature dependence of absorption; see Materials and Methods). Equation 5 shows that longwave cooling sensitivity is the product of two factors. The first is thermodynamic: All else being equal, longwave cooling at all wave numbers increases in proportion to the relative rate at which water vapor is added to the atmosphere ($\frac{d\text{ln}\mathrm{WVP}}{{\mathrm{dT}}_{s}}$, i.e., Clausius-Clapeyron). The second is spectral: Longwave cooling sensitivity is largest at wave numbers where surface Planck emission [$B_{\nu }\left(T_{s}\right)$] is substantial, the optical depth of water vapor is near unity ($\tau_{\nu ,\text{wv}}e^{-\tau_{\nu ,\text{wv}}}$ is largest where $\tau_{\nu ,\text{wv}}∼1$), and the optical depth of non-condensable emitters is sufficiently small ($\tau_{\nu ,o}\ll 1$).

To make more concrete predictions, we adopt idealized models of the spectroscopy of water vapor (*24*, *25*) and carbon dioxide (*25*, *26*). Spectrally variable line absorption by water vapor is assumed to have an exponential relationship between mass absorption coefficient and wave number in its two main longwave absorption bands: the mid-infrared band (dominated by vibrational-rotational transitions, for which absorption peaks at 1500 cm^–1^) and the far-infrared band (dominated by rotational transitions, for which absorption peaks at 150 cm^–1^). The spectral region in between these features, the water vapor window, contains only weak absorption due to the water vapor continuum, which we assume to be spectrally invariant (*25*). Carbon dioxide, Earth’s primary non-condensable emitter in the longwave, is assumed to have a mass absorption coefficient that falls off exponentially in wave number from its peak value at 667 cm^–1^.

Calculations below explore the implications of this spectroscopy in idealized atmospheric columns broadly consistent with tropospheric radiative-convective equilibrium (*27*, *28*): Relative humidity is a constant 70%, and the temperature follows a moist adiabat from the surface to a fixed temperature at the tropopause (220 K). Stratospheric humidity falls off vertically in accordance with the same moist adiabat, while stratospheric temperature is held vertically constant at its value at the tropopause (220 K) (*23*–*26*). We compare our idealized model to full-physics, line-by-line calculations from the Atmospheric Radiative Transfer Simulator (*29*) in the same idealized atmosphere.

**Fig. 1.**
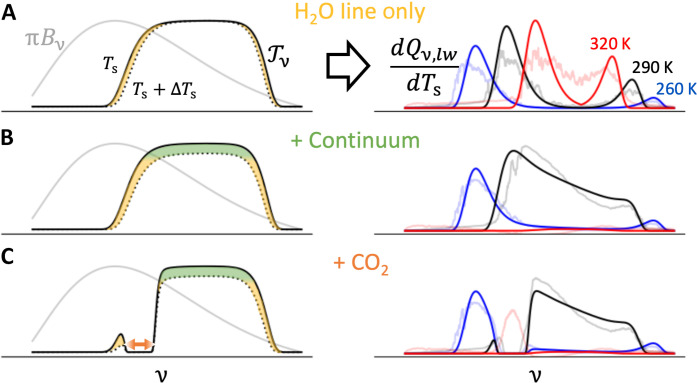
The radiative mechanisms underlying hydrological sensitivity. Via Eq. 4, the product of Planck emission (gray, left) and the change in atmospheric transmission with surface warming (difference between solid and dotted black lines, left) yield the column-integrated longwave cooling sensitivity (solid lines, right). Under line absorption by water vapor (**A**), the water vapor window closes from its edges (yellow), so this is where cooling sensitivity is greatest. Continuum absorption (**B**) closes the water vapor window across its entirety (green), increasing cooling sensitivity at all wave numbers until the window entirely closes. Carbon dioxide (**C**) masks cooling sensitivity where it is optically thick (orange). Our spectral model (solid lines, right) compares reasonably well to full-physics, line-by-line calculations (faded lines, right) across a range of surface temperatures (260, 290, and 320 K, cool to warm colors) and wave numbers (150 to 1450 cm^–1^).

Figure 1 schematically illustrates the relationships between spectroscopy, thermodynamics, and hydrological sensitivity. Spectrally resolved longwave cooling sensitivity is largest in spectral regions where the water vapor window (the set of wave numbers with substantial transmission) closes (that is, transitions from optically thin to optically thick). Two absorption mechanisms, with very different characteristics, contribute to the closing of the water vapor window. Spectrally variable absorption by water vapor lines (Fig. 1, top row) causes the water vapor window to close from the edges; these spectral regions are the largest contributors to column-integrated cooling.

Absorption by the water vapor continuum (Fig. 1, middle row), however, causes the water vapor window to close across its entirety once surface temperature and the resulting water vapor path are sufficiently high. This causes longwave cooling sensitivity to have a notably different dependence on surface temperature. At low surface temperatures, continuum absorption is largely negligible. At higher surface temperatures, however, continuum absorption causes even the most transparent wave numbers in the water vapor window to have order unity optical depth such that transmission and cooling are sensitive to surface temperature across the entire water vapor window. At the highest surface temperatures considered, the water vapor window closes entirely, and the change in spectrally resolved cooling with surface temperature is near zero at all wave numbers.

Non-condensable greenhouse gases such as carbon dioxide mask spectral windows (*30*–*32*), suppressing the hydrological response to warming at most Earth-like surface temperatures (Fig. 1, bottom row). For surface temperatures below about 310 K, column-integrated cooling is insensitive to surface temperature in spectral regions where CO_2_ is optically thick (see the Supplementary Text). Similar mechanisms determine the magnitude of a direct response $\Delta Q_{\text{direct}}$ caused by a change in CO_2_ concentration. As CO_2_ concentration increases in the absence of any change in surface temperature, it masks more tropospheric emission by water vapor, reducing column-integrated longwave cooling and, thus, mean rainfall.

Similar physics are at play in the shortwave. Only water vapor absorbs substantially in the near-infrared ($\tau_{\nu ,o}=0$), so the change in column-integrated shortwave heating with surface temperature is

$$\frac{dH_{\nu ,\text{sw}}}{{\mathrm{dT}}_{s}}=I_{\nu }\frac{d\text{ln}\mathrm{WVP}}{{\mathrm{dT}}_{s}}\tau_{\nu ,\text{wv}}e^{-\tau_{\nu ,\text{wv}}}$$ (6)

Shortwave heating, like longwave cooling, increases most rapidly when $\tau_{\nu ,\text{wv}}\approx 1$. As the surface warms, absorption features, windows in the near-infrared, transition from optically thin to optically thick as tropospheric water vapor increases. This increases the amount of sunlight that the atmosphere absorbs at those wave numbers, muting the hydrological response.

### Quantitative understanding: Magnitude and surface temperature dependence

To understand more quantitatively how spectroscopy and radiative transfer set the magnitude of hydrological sensitivity and its dependence on surface temperature, we estimate the broadband (spectrally integrated) change in cooling with surface temperature $\frac{dQ}{{\mathrm{dT}}_{s}}$ across an increasingly complete set of absorption mechanisms.

Line absorption by water vapor is the dominant mechanism producing radiative cooling in the troposphere (*24*). The amount of light that water vapor lines absorb per unit mass of water vapor falls off roughly inverse-exponentially with wave number toward the center of the water vapor window, while water vapor burden increases quasi-exponentially due to thermodynamic constraints. These effects largely cancel, causing the water vapor window to close at a near constant rate with surface temperature. An estimate of $\frac{dQ}{{\mathrm{dT}}_{s}}$ due to longwave line absorption by water vapor can be obtained by approximating the spectral integral of Eq. 5 across each absorption band (*24*) while neglecting absorption by the water vapor continuum and non-condensable gases (see Materials and Methods). This yields

$$\frac{dQ_{\text{lw},\text{wv}}^{\text{line}}}{{\mathrm{dT}}_{s}}=\alpha \pi \sum_{j}^{2}B_{\nu }\left(\nu_{1,j},T_{s}\right)l_{j}$$ (7)

where the index j denotes either the far- or mid-infrared absorption band. In band j, $\nu_{1,j}$ is the wave number for which $\tau_{\nu ,\text{wv}}=1$, and $l_{j}$ is the spectral width over which the absorption coefficient decays by a factor of *e* away from its peak. The thermodynamic constraint $\frac{d\text{ln}\mathrm{WVP}}{{\mathrm{dT}}_{s}}$ (i.e., Clausius-Clapeyron) is approximated as a constant α. The specific analytical approximations and spectroscopic parametrizations used to derive Eq. 7 (and Eqs. 8 and 9 to follow) can be found in Eqs. 17 to 22 in Materials and Methods.

For a given optical depth, the rate at which emissivity increases with surface temperature is $\frac{d\epsilon_{\nu ,\text{wv}}}{{\mathrm{dT}}_{s}}={\alpha \tau }_{\nu ,\text{wv}}e^{-\tau_{\nu ,\text{wv}}}$. This maximizes at $\frac{\alpha }{e}$, when $\tau_{\nu ,\text{wv}}=1$. Thus, $\frac{\alpha }{e}$ is the “emissivity gradient at unity optical depth,” describing the rate at which atmospheric emissivity increases with surface temperature at wave numbers with $\tau_{\nu ,\text{wv}}=1$, and $l_{j}e$ a measure of band *j*’s “spectral proximity to unity optical depth,” indicating how collectively close the wave numbers in band *j* are to unity optical depth (*24*) (or, more precisely, how many wave numbers with $\tau_{\nu ,\text{wv}}=1$ are needed to achieve a spectrally integrated emissivity gradient equal to that of band j). The emissivity gradient at unity optical depth ($\frac{\alpha }{e}$) is a function of water vapor’s thermodynamic properties (i.e., how quickly surface warming adds water vapor to the atmosphere); spectral proximity to unity optical depth ($l_{j}e$) is a function of water vapor’s spectroscopic properties (i.e., how many wave numbers in band j substantially increase atmospheric emissivity when an increment of water vapor is added). Both are constant, so surface Planck emission alone sets the surface temperature dependence of column-integrated broadband cooling sensitivity by water vapor lines.

**Fig. 2.**
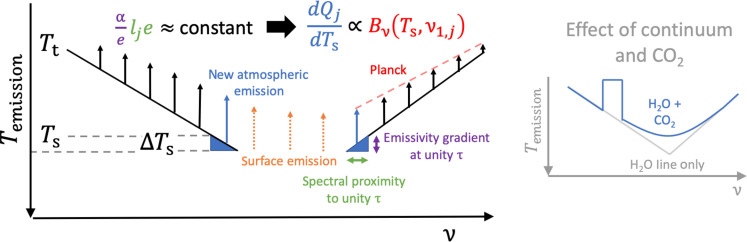
Planck emission sets the surface temperature dependence of $\frac{dQ_{\text{lw},\text{line}}}{{\mathrm{dT}}_{s}}$. Emission temperature $T_{\text{emission}}$, shown in black, varies from the tropopause temperature ($T_{t}$) in the optically thickest regions to the surface temperature ($T_{s}$) in optically thin regions. Surface warming does not alter existing atmospheric emission (black arrows) but rather exchanges surface emission (orange arrows) for added atmospheric emission (blue arrows) in the spectral regions where $\tau_{\nu ,\text{wv}}\approx 1$. The emissivity gradient at unity optical depth ($\frac{\alpha }{e}$, purple) is set by Clausius-Clapeyron, and the spectral proximity to unity optical depth ($l_{j}e$, green) is set by the spectroscopy of water vapor. These are constant, making the added atmospheric emission (blue) in each band *j* proportional to Planck emission (red) at $T_{s}$ and $\nu_{1,j}$. Right: How the water vapor continuum and CO_2_ modify $T_{\text{emission}}$.

These ideas are illustrated in the left panel of Fig. 2. In the optically thickest part of the atmosphere, emission to space occurs at a temperature close to the tropopause temperature ($T_{t}$); in optically thin regions, emission is dominated by the surface. Surface warming does not change atmospheric emission at a given temperature level (*14*, *15*) but instead extends atmospheric emission to warmer temperature levels (*8*) by increasing column-integrated water vapor. This added emission occurs in spectral regions where $\tau_{\nu ,\text{wv}}\approx 1$, that is, where the water vapor window closes. Surface warming closes the water vapor window at a constant rate ($\frac{\alpha }{e}l_{j}e$ is constant), so only the Planck function depends on surface temperature. This makes the added atmospheric emission in each band j proportional to Planck emission at $T_{s}$ and $\nu_{1,j}$.

Water vapor lines cause atmospheric emission to grow at a near constant rate with surface temperature. This is because the exponential increase in water vapor path with surface temperature is balanced by the exponential decrease in absorption coefficient with wave number. Continuum absorption breaks this symmetry. The change in column-integrated longwave cooling with surface temperature for a water vapor atmosphere including line and continuum absorption can be approximated by

$$\frac{dQ_{\text{lw},\text{wv}}}{{\mathrm{dT}}_{s}}=\frac{dQ_{\text{lw},\text{wv}}^{\text{line}}}{{\mathrm{dT}}_{s}}e^{-\tau_{c}}\left[1-\text{Ei}\left(\tau_{c}-\tau_{\text{min}}\right)\tau_{c}\right]$$ (8)

where $\tau_{c}$ is the continuum’s contribution to the atmospheric optical depth, Ei(x) is the exponential integral, and $\tau_{\text{min}}$ is the optical depth at the optically thinnest wave number in the water vapor window (see Materials and Methods). Equation 8 implies that column-integrated cooling sensitivity, and, by extension, hydrological sensitivity, peaks when the optical depth of the (gray) continuum is ~1 {$e^{-\tau_{c}}\left[1-\text{Ei}\left(\tau_{c}-\tau_{\text{min}}\right)\tau_{c}\right]$ maximizes when $\tau_{c}\approx 1$}. At 70% relative humidity, this occurs at a surface temperature of ~300 K, a value comparable to figure 4 of (*8*). Equation 8 also implies that hydrological sensitivity reverses sign at about 320 K, when the optical depth of the continuum is ~3 and $e^{-\tau_{c}}\approx 0$. Comprehensive Earth-system models robustly produce a peak in rainfall at 320 to 330 K regardless of the forcing agent (*18*). This suggests that continuum absorption sets the temperature at which hydrological sensitivity reverses sign in hothouse climates; once longwave atmospheric cooling is capped, shortwave heating by water vapor, which continues to increase with surface temperature, dominates the hydrological response.

Changing (vertically constant) relative humidity has almost no effect on longwave cooling from line absorption (*24*) or its sensitivity to surface temperature. However, relative humidity plays a central role in setting the peak and fall-off in cooling sensitivity when continuum absorption is included. At higher relative humidity, $\tau_{c}$ exceeds any given value, and, thus, sensitivity both peaks and changes sign, at a cooler surface temperature (see the Supplementary Text).

Non-condensable greenhouse gases such as carbon dioxide have a masking effect (*31*, *32*), reducing the sensitivity of cooling to surface temperature in spectral regions where they are optically thick. We represent this impact by assuming the sensitivity of cooling to surface temperature is zero at wave numbers where the optical depth of carbon dioxide exceeds unity. For a CO_2_ concentration of 400 parts per million (ppm), this masked spectral region ranges from about 742 to 593 cm^–1^. Modifying Eq. 8 to include these physics yields

$$\frac{dQ_{\text{lw},\text{wv}+c}}{{\mathrm{dT}}_{s}}=\frac{dQ_{\text{lw},\text{wv}}}{{\mathrm{dT}}_{s}}-\alpha \pi B_{\nu }\left(\nu_{1,f},T_{s}\right)l_{f}\Phi_{\text{low}}^{\text{high}}$$ (9)

where $\Phi =\left[e^{-\tau_{\text{wv}}}-\tau_{c}e^{-\tau_{c}}\text{Ei}\left(-\tau_{\text{wv}}+\tau_{c}\right)\right]$ is evaluated on the high (742 cm^–1^) and low (593 cm^–1^) wave number side of the CO_2_ band (see Materials and Methods). The latter term in Eq. 9 approximates the size of the spectral bite that carbon dioxide takes out of the far-infrared band’s (labeled with the subscript f) cooling response to surface temperature. The temperature dependence induced by CO_2_, thus, depends on the extent to which water vapor’s most sensitive wave numbers (i.e., those near $\tau_{\nu ,\text{wv}}=1$) overlap with the masked spectral region; the greater the overlap, the more the spectral proximity to unity optical depth is reduced. Continuum absorption accelerates, and carbon dioxide masks, the closing of the water vapor window under surface warming. These effects, by extension, affect hydrological sensitivity. They are shown schematically in the right panel of Fig. 2.

We can approximate the direct response to changes in CO_2_, in particular, by again assuming that such changes primarily mask tropospheric emission by water vapor. This yields

$${\left.\Delta Q_{\text{direct}}=-2\text{ln}\left(\frac{q_{f}}{q_{i}}\right)l_{0}\pi \left[B_{\nu }\left(T_{\text{em}}\right)-B_{\nu }\left(T_{s}\right)e^{-\tau_{\text{wv}}}\right]\right|}_{\nu_{0}}$$ (10)

where $\nu_{0}$ is the wave number at the center of the CO_2_ band, $l_{0}$ is the spectral width over which the absorption coefficient of CO_2_ decays by a factor of *e* away from its peak, and $q_{i}$ and $q_{f}$ are the initial and final CO_2_ concentrations, respectively. $T_{\text{em}}$ is the “masked” emission temperature at $\nu_{0}$, that is, the emission temperature that would be observed at $\nu_{0}$ in the absence of CO_2_ (*26*). Like the instantaneous tropopause forcing due to changes in CO_2_ (*26*), Eq. 10 approximates how much additional emission CO_2_ masks when its concentration is changed. Crucially, however, Eq. 10 excludes masked surface emission because this does not affect the tropospheric energy budget. (The Supplementary Text derives and evaluates the accuracy of Eq. 10 using line-by-line calculations.)

Shortwave heating via absorption by water vapor, which damps hydrological sensitivity, occurs primarily in the near-infrared, where water vapor has several partly transparent windows. We approximate the collective effect of these regions with a single window (see Materials and Methods). This approach does not capture the spectral behavior of $\frac{dH_{\nu ,\text{sw}}}{{\mathrm{dT}}_{s}}$ but does describe the aggregate effect of shortwave heating on the hydrological response to warming. Adding this damping to Eq. 9 yields

$$\frac{dQ}{{\mathrm{dT}}_{s}}=\frac{dQ_{\text{lw},\text{wv}+c}}{{\mathrm{dT}}_{s}}-\frac{dH_{\text{sw}}}{{\mathrm{dT}}_{s}}=\frac{dQ_{\text{lw},\text{wv}+c}}{{\mathrm{dT}}_{s}}-\alpha I_{\nu }\left(\nu_{1,n}\right)l_{n}$$ (11)

where the subscript n refers to the near-infrared band. Equation 11 is derived using a similar set of analytical approximations and spectroscopic parametrizations as those used to derive Eqs. 7 to 9 (see Eq. 23 in Materials and Methods for more details).

There are several parallels with the longwave. For one, we can define an “absorptivity gradient at unity optical depth” ($\frac{\alpha }{e}$) which, like the emissivity gradient at unity optical depth, is constant and purely a function of water vapor’s thermodynamic properties. Similarly, as with line absorption in the mid- and far-infrared bands, water vapor’s absorption coefficient decays exponentially with wave number in the near-infrared, causing its spectral proximity to unity optical depth ($l_{n}e$) to be constant as well. Thus, the temperature dependence of shortwave heating sensitivity is set by the wave number dependence of the incident solar flux [$I_{\nu }\left(\nu_{1,n}\right)$]. Because $\nu_{1,n}$ grows linearly with $T_{s}$ and incident solar flux increases with wave number in the near-infrared, column-integrated shortwave heating sensitivity grows accordingly with surface temperature (*8*).

**Fig. 3.**
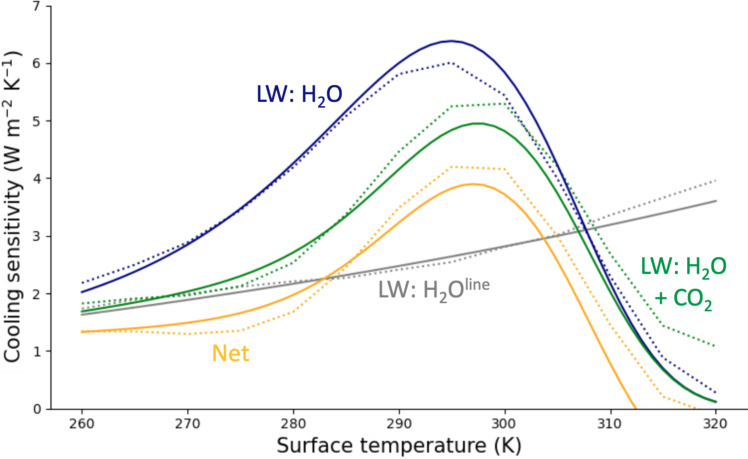
Sensitivity of column-integrated radiative cooling to changes in surface temperature under a hierarchy of different physical assumptions. Longwave (LW) line absorption by water vapor (gray), longwave line and continuum absorption by water vapor (blue), longwave absorption by water vapor and carbon dioxide (green), and net absorption (longwave and shortwave) by water vapor and carbon dioxide (yellow) for a zenith angle of 75° (reflective of global average insolation). Solid lines show results from Eqs. 7 to 9 and 11. Dotted lines show results from the full-physics, line-by-line radiative transfer model ARTS.

The estimates in Eqs. 7 to 9 and 11 capture the magnitude and surface temperature dependence of column-integrated radiative cooling sensitivity as computed by a full-physics line-by-line model (Fig. 3). Below 290 K, longwave line absorption by water vapor (gray) is a rough but useful proxy for cooling sensitivity (orange). Equation 8 broadly captures the peak and sign-reversal in cooling sensitivity, although these features are slightly shifted when CO_2_ absorption and shortwave heating are included. Equations 9 and 11 largely capture the magnitude of masking by CO_2_ and muting by shortwave heating, although the neglect of increases in tropospheric emission by non-condensable gases under warming degrades the validity of the model past about 310 K and underestimates the surface temperature at which $\frac{dQ}{{\mathrm{dT}}_{s}}$ vanishes (see the Supplementary Text).

### Sensible heating and clouds

Our estimates of the sensitivity of column-integrated atmospheric cooling to surface temperature (Eqs. 7 to 9 and 11) are applicable only in clear skies. Much of the earth, however, is cloudy. Clouds are white, or nearly so, meaning that the spectral variation of cloud opacity is modest; most clouds are also optically thick in the infrared and, hence, mask any cooling to space from below the cloud top. In areas in which cloud-top temperature is independent of surface temperature, i.e., for deep clouds in the tropics (*33*, *34*), cooling to space is thus largely independent of surface temperature (neglecting pressure dependence and changes in relative humidity above the cloud). In this sense, the presence of deep clouds acts to suppress changes in mean rainfall with surface temperature. In regions in which cloud top pressures are controlled by factors other than surface temperature, for example, over low clouds in the subtropics (*35*), cloud tops play the same role in determining cooling to space as the surface does in clear skies. Thus, neglecting changes in surface exchanges, the sensitivity of cooling can be approximated using the cloud-top temperature in place of surface temperature.

In addition, Eq. 1 is useful for understanding precipitation changes with surface temperature only to the extent that sensible heating, the other component of the atmospheric energy budget, does not change with surface temperature. However, changes in sensible heating may have been roughly as large as changes in latent heating in the recent past (*36*) and are an important factor contributing to differences among climate models (*37*). Changes in sensible heating may be addressed by assuming that the free atmosphere, where radiative cooling is balanced primarily by condensational heating, is distinct from the planetary boundary layer, where radiative cooling is balanced primarily by sensible heating (*38*, *39*). This division neglects any dry static energy fluxes from the boundary layer into the free atmosphere, although these are thought to be small in the global average (*7*). With this simplification, hydrological sensitivity is determined by the column-integrated radiative cooling sensitivity of the free atmosphere ($\frac{dQ_{\text{free}}}{{\mathrm{dT}}_{s}}$) rather than the total atmosphere. If the temperature at the bottom of the free atmosphere $T_{f}$ is sufficiently close to the surface temperature, the upward flux produced by the surface and the atmosphere below $T_{f}$ is roughly equal to $\pi B_{\nu }\left(T_{f}\right)$, the flux that would be produced by an imaginary surface at $T_{f}$ (see the Supplementary Text). This equivalence yields a refined estimate of hydrological sensitivity given by

$$L\Delta P\approx \Delta Q=\left({\frac{dQ}{{\mathrm{dT}}_{s}}|}_{T_{s}=T_{f}}\right)\frac{{\mathrm{dT}}_{f}}{{\mathrm{dT}}_{s}}\Delta T_{s}+\Delta Q_{\text{direct}}$$ (12)

Because clear-sky hydrological sensitivity is controlled by changes in free (rather than total) atmospheric transmission, sensible heating and shallow clouds can be thought of as acting to decrease the surface temperature at which hydrological sensitivity is evaluated (*8*). Boundary layers are typically 5 to 10 K deep; temperatures at the tops of shallow clouds vary more widely. Because $\frac{{\mathrm{dT}}_{f}}{{\mathrm{dT}}_{s}}$ is typically greater than one, sensible heating also slightly amplifies hydrological sensitivity, although this effect is minimal (about 10%). (The Supplementary Text evaluates the accuracy of Eq. 12 using line-by-line calculations.) In the absence of changes in surface temperature, sensible heat fluxes should not notably change, so the direct response is unmodified.

### Comparison with comprehensive GCM simulations

Our analytical model uses the material properties of Earth’s main greenhouse gases to explain how column-integrated tropospheric emission changes with surface temperature and CO_2_ concentration. The results, especially the prediction of a maximum in hydrological sensitivity at high surface temperatures, are consistent with simulations with comprehensive general circulation models (GCMs).

**Fig. 4.**
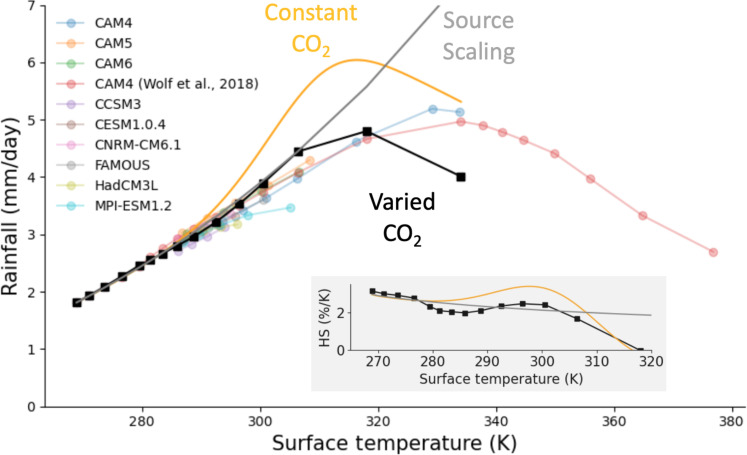
Atmospheric spectroscopy controls sensitivity of mean rainfall to surface temperature. We compare a variety of GCMs [faded lines; (*40*)] to predictions from our pen-and-paper model under a constant CO_2_ concentration of 400 ppm (orange, Eq. 12) and under varied CO_2_ concentration [black, Eq. 12 and Eq. 10, using a climate sensitivity given by Wolf *et al*. (*41*)]. The gray line shows the prediction from the source scaling (Eq. 13). Inset: The percent change in mean rainfall with surface temperature as predicted by these same analytical models (same color labels). HS, hydrological sensitivity.

Figure 4 compares simulations of numerous doublings and halvings of CO_2_ from preindustrial levels taken from (*40*) (faded lines, their Fig. 1C) to our pen-and-paper model, which we integrate forward in surface temperature and CO_2_ concentration from the GCM-derived precipitation at the coolest surface temperature. Varying surface temperature and CO_2_ concentration in tandem requires some choice for climate sensitivity, which we take from (*41*)’s simulations (red faded lines), as these span the largest range of surface temperatures and CO_2_ concentrations. Different physics control the hydrological cycle in hothouse climates (*18*), so we only consider surface temperatures up to about 335 K. Because GCM surface temperature and CO_2_ concentrations covary here, total changes in precipitation include both the response to warming ($\frac{dQ}{{\mathrm{dT}}_{s}}\Delta T_{s}$) and the direct response to changes in CO_2_ ($\Delta Q_{\text{direct}}$); these are sometimes called the slow and fast responses of precipitation (*42*, *43*).

Under constant CO_2_ (orange, 400 ppm), the hydrological response is driven entirely by changes in surface temperature (Eq. 12). This response is generally greater than the hydrological response under varied CO_2_ conditions (black) because it excludes the reduction in tropospheric cooling that directly results from increases in CO_2_. At surface temperatures below 290 K, the direct response is small compared to the temperature driven response because CO_2_ primarily masks emission by the surface, not the troposphere. Above 290 K, however, water vapor is sufficiently optically thick in the CO_2_ band that the hydrological response is no longer well approximated by Eq. 12 alone.

Below 320 K, our pen-and-paper model falls within or quite near more comprehensive GCM simulations, suggesting that the physics not represented in our simple model, most notably cloud radiative effects and spatial variations in surface temperature and relative humidity, are second-order effects in this temperature range. Above 320 K, however, our model substantially underpredicts global mean rainfall. While this might be the result of a break down in the clear-sky, single column approximation, it also could be due to unaccounted-for increases in tropospheric emission by CO_2_, particularly within the 1000 cm^–1^ absorption band, which was entirely neglected in this study, or due to a breakdown in the radiative constraint on mean rainfall more broadly (*8*). Deviations from Simpson’s law may also play a crucial role, perhaps partially explaining why hydrological sensitivity reverses sign in CAM4 at a somewhat warmer surface temperature (330 K) than our simple analysis of the continuum might suggest (320 K).

Figure 4 also includes a “source-scaling” (gray), which approximates the hydrological response when the direct response is small compared to the temperature-driven response and when the spectral proximity to unity optical depth is roughly constant across each of water vapor’s absorption bands in the longwave and shortwave. Under these conditions, hydrological sensitivity varies only with the “sources” of additional longwave cooling [$\sum_{j}^{2}B_{\nu }\left(\nu_{1,j},T_{s}\right)$] and shortwave heating [$I_{\nu }\left(\nu_{1,n}\right)$] by water vapor. Because longwave emission by the mid-infrared band and shortwave absorption by the near-infrared band largely cancel, apparent hydrological sensitivity grows primarily with longwave emission in the far-infrared band, where $\frac{\partial \text{ln}B_{\nu }}{\partial \text{ln}T}\approx 4$ at the wave numbers near unity optical depth (*24*). This implies that $\frac{\Delta P}{\Delta T_{s}}\propto T_{s}^{4}$, such that logarithmic hydrological sensitivity is given by

$$\frac{\left(\frac{\Delta P}{\Delta T_{s}}\right)}{P}\approx \frac{T_{s}^{4}}{\int_{T_{t}}^{T_{s}}T_{s}^{\prime 4}{\mathrm{dT}}_{s}^{\prime }}\approx \frac{5T_{s}^{4}}{T_{s}^{5}-T_{t}^{5}}$$ (13)

The value of the source scaling of mean rainfall with surface temperature, ~2%/K (Fig. 4, inset), is broadly consistent with estimates emerging from simulations with dynamical models over a wide range of surface temperatures (270 to 300 K). Below about 290 K, this agreement appears robust. Here, the fundamental approximations used to derive the source scaling are largely valid: The direct response is small compared to the temperature-driven response, and the spectral proximity to unity optical depth is roughly constant in each of water vapor’s absorption bands, varying by no more than 20%. Agreement beyond 290 K, however, is primarily the result of a compensation between the continuum and the direct response (Fig. 4, inset). This compensation reduces if not outright eliminates the peak in hydrological sensitivity implied by Eq. 8, perhaps explaining why apparent hydrological sensitivity is nearly constant over such a wide range of CO_2_ forcings (*17*). The source scaling of mean rainfall may also offer some physical intuition for why mean rainfall grows roughly quadratically with the temperature depth of the troposphere (*8*); Eq. 13 implies $P\propto T_{s}^{5}-T_{t}^{5}$, which appears roughly quadratic in $T_{s}-T_{t}$ over Earth-like surface temperatures.

## DISCUSSION

Our spectroscopic theory, much of which is a synthesis of existing work, elucidates several robust yet to date unexplained features of the global hydrological cycle. Changes in column-integrated radiative cooling, a proxy for mean rainfall, are governed primarily by changes in atmospheric opacity. When atmospheric opacity increases at a constant rate with surface warming, as is largely the case for surface temperatures less than 290 K, integrated cooling grows primarily with longwave emission in the far-infrared band, implying a present-day hydrological sensitivity of about 2%/K (*3*–*5*). Apparent hydrological sensitivity remains roughly 2%/K over numerous doublings of CO_2_ concentration (*17*, *40*), due, in large part, to a cancellation between continuum absorption and the direct response due to carbon dioxide. Once the continuum’s optical depth exceeds about three, however, the water vapor window fully closes, and, with shortwave heating by water vapor continuing to increase with surface temperature, hydrological sensitivity reverses sign at about 320 K (*18*, *40*).

At Earth-like surface temperatures, the magnitude of hydrological sensitivity, about 2%/K, is notably smaller than the roughly 7%/K growth of column-integrated water vapor path with surface temperature predicted by the Clausius-Clapeyron relationship (*4*). At first glance, this seems to suggest that hydrological sensitivity is untethered from thermodynamic constraints. Clausius-Clapeyron is closely tied to hydrological sensitivity; that the latter grows more slowly than the former emerges from the additional constraints imposed by water vapor’s spectroscopy. In an optically thin grey atmosphere the change in transmission with surface temperature would be proportional to water vapor path ($-\frac{dT}{{\mathrm{dT}}_{s}}\approx \frac{d\tau }{{\mathrm{dT}}_{s}}\propto \mathrm{WVP}$), causing column-integrated cooling to grow roughly with Clausius-Clapeyron (actually somewhat faster due to the additional impact of Planck emission; see the Supplementary Text). Similar physics arise in the real atmosphere at surface temperatures near that of the tropopause, when nearly all of water vapor’s wave numbers have optical depth less than unity and thus radiative cooling changes rapidly (in relative terms) with surface temperature (*24*). At surface temperatures this cold, however, the radiative constraint on mean rainfall breaks down.

In a single column, the sensitivity of clear-sky free-atmospheric radiative cooling to changes in surface temperature depends on the free-atmospheric water vapor path, the temperature at the bottom of the free atmosphere, and the sensitivity of these two quantities to changes in surface temperature. The sensitivity of globally integrated precipitation to global-mean surface temperature (under clear skies), therefore, depends on the distribution of these conditions and the way in which this covaries with a given change in global-mean surface temperature. The variation in hydrological sensitivity across climate models is, thus, likely due to differences in these distributions and in the distribution of clouds and how these clouds change with warming. This also suggests that real-world hydrological sensitivity could be more tightly constrained by observations, subject to uncertainty in how free-atmospheric water vapor path and the temperature at the bottom of the free atmosphere change with global-mean surface temperature.

## MATERIALS AND METHODS

This section details the development of the conceptual models encapsulated in Eqs. 2 to 11. The Supplementary Text provides numerical values for all model parameters, and all data used in constructing this study’s figures can be found at https://zenodo.org/records/13293290.

Writing the spectrally resolved column-integrated longwave heating as the difference between net (downward minus upward) flux at the top of the atmosphere and the surface and regrouping like terms allows us to write the cooling as the sum of cooling to space and surface exchange terms

$$Q_{\nu ,\text{lw}}=\int_{0}^{\tau_{\nu }}\pi B_{\nu }\left(\tau \right)e^{-\tau }d\tau -\int_{0}^{\tau_{\nu }}\pi \left[B_{\nu }\left(T_{s}\right)-B_{\nu }\left(\tau \right)\right]e^{-\left(\tau_{\nu }-\tau \right)}d\tau$$ (14)

where $\tau_{\nu }$ is the column optical depth. Neglecting changes in surface exchanges with surface temperature (an assumption assessed in the Supplementary Text) yields

$$\frac{dQ_{\nu ,\text{lw}}}{{\mathrm{dT}}_{s}}=\pi B_{\nu }\left(\tau_{\nu }\right)e^{-\tau_{\nu }}\frac{d\tau_{\nu }}{{\mathrm{dT}}_{s}}+\int_{0}^{\tau_{\nu }}\pi \frac{d}{{\mathrm{dT}}_{s}}\left[B_{\nu }\left(\tau \right)e^{-\tau }\right]d\tau$$ (15)

where we have used Leibniz’s rule to expand the cooling-to-space term. Equation 4 follows by assuming the air temperature at the surface is equal to the surface temperature [$B_{\nu }\left(\tau_{\nu }\right)=B_{\nu }\left(T_{s}\right)$] and the atmospheric temperature at a given optical depth does not change with surface temperature [$\frac{{\mathrm{dB}}_{\nu }\left(\tau \right)}{{\mathrm{dT}}_{s}}=0$] (*14*, *15*, *44*).

To link the spectral properties of the atmosphere to its thermal structure and composition, we express column optical depth as the sum of contributions from carbon dioxide and water vapor, the latter of which absorbs through spectrally dependent lines and a spectrally independent (gray) continuum

$$\tau_{\nu }=\tau_{\nu ,\text{wv}}+\tau_{\nu ,o}=D\left[\left(k_{\nu ,\text{wv}}^{\text{line}}+k_{c}\right)WVP+\frac{k_{\nu ,\text{cd}}p_{s}\rho }{2g}\right]$$ (16)

Here, *g* is the acceleration due to gravity; $p_{s}$ is the surface pressure; ρ is the mass concentration of CO_2_; $k_{\nu ,\text{cd}}$ is the mass absorption coefficient of carbon dioxide; $k_{\nu ,\text{wv}}^{\text{line}}$ and $k_{c}$ are the mass absorption coefficients of water vapor due to lines and continuum absorption, respectively; and *D* is a diffusivity factor to approximate the angularly integrated fluxes (*45*). Equations 5 and 6 follow after assuming that only WVP depends on surface temperature.

We parameterize line absorption by water vapor in the far- and mid-infrared bands by assuming the absorption coefficient within each band decreases exponentially with wave number away from a peak value and spectral location (*24*, *25*). This yields

$$k_{\nu ,\text{wv}}^{\text{line}}=\text{max}\left(\left\{\begin{array}{c} k_{f}e^{\left[-\frac{\left(\nu -\nu_{f}\right)}{l_{f}}\right]}\\ k_{m}e^{\left[-\frac{\left(\nu_{m}-\nu \right)}{l_{m}}\right]} \end{array}\right.\right)$$ (17)

where the subscripts f and m refer to the far- and mid-infrared bands, respectively.

In the shortwave, we neglect absorption by CO_2_ and parameterize water vapor’s total (line and continuum) absorption in the near-infrared as

$$k_{\nu ,\text{wv}}=k_{n}e^{-\frac{\nu }{l_{n}}}$$ (18)

Parameters $k_{n}$, $l_{n}$, and $k_{c}$ are chosen by minimizing the least squared error between the parameterization of the mass absorption coefficient of water vapor and that computed by atmospheric radiative transfer simulator (ARTS) at standard temperature and pressure.

Broadband results (Eqs. 7 to 9) are obtained by integrating Eq. 5 over all wave numbers in the longwave. This yields

$$\frac{dQ_{\text{lw}}}{{\mathrm{dT}}_{s}}=\frac{d\text{ln}\mathrm{WVP}}{{\mathrm{dT}}_{s}}\int_{0}^{\infty }e^{-\tau_{\nu ,o}}\pi B_{\nu }\left(T_{s}\right)\tau_{\nu ,\text{wv}}e^{-\tau_{\nu ,\text{wv}}}d\nu$$ (19)

which can be evaluated under various physical assumptions. We approximate this spectral integral by making two key simplifying assumptions. First, we distinguish between spectral regions where CO_2_ is optically thin ($\tau_{\nu ,o}<1$), where we neglect carbon dioxide entirely, and regions where CO_2_ is optically thick ($\tau_{\nu ,o}>1$), where we assume complete masking by carbon dioxide such that $\frac{dQ_{\nu ,\text{lw}}}{{\mathrm{dT}}_{s}}=0$. Second, because the Planck function varies slowly with wave number in comparison to $\tau_{\nu ,\text{wv}}e^{-\tau_{\nu ,\text{wv}}}$, we evaluate $B_{\nu }\left(T_{s}\right)$ at $\nu_{1,j}$, the wave number in band j where $\tau_{\nu ,\text{wv}}=1$, that is, where $\tau_{\nu ,\text{wv}}e^{-\tau_{\nu ,\text{wv}}}$ peaks (*24*). Extracting this term from the spectral integral yields

$$\frac{dQ_{\text{lw}}}{{\mathrm{dT}}_{s}}=\alpha \pi \sum_{j}^{2}B\left(\nu_{1,j},T_{s}\right)\left(\int \tau e^{-\tau }d\nu_{j}-\int \tau e^{-\tau }d\nu_{c,j}\right)$$ (20)

where $\int d\nu_{j}$ is the integral over all wave numbers in band *j* and $\int d\nu_{c,j}$ is the integral over the wave numbers in band j for which carbon dioxide has an optical depth greater than one. We also let $\frac{d\text{ln}\mathrm{WVP}}{{\mathrm{dT}}_{s}}=\alpha$ (a constant) and write $\tau_{\nu ,\text{wv}}$ as τ for brevity. In all cases $\nu_{1,j}$ is defined as the wave number in band j where τ=1 assuming line absorption by water vapor alone. This neglects the minor impact of the continuum on the choice of the wave number at which the Planck function is evaluated.

To analytically evaluate Eq. 20, we take the antiderivative of $\tau e^{-\tau }$ in each of water vapor’s absorption bands. Invoking the parameterization for water vapor’s spectroscopy, we have

$$\int \tau e^{-\tau }d\nu_{f}=l_{f}\left[e^{-\tau }-\tau_{c}e^{-\tau_{c}}\text{Ei}\left(-\tau +\tau_{c}\right)\right]+C_{f}$$ (21)

in the far-infrared band and

$$\int \tau e^{-\tau }d\nu_{m}=-l_{m}\left[e^{-\tau }-\tau_{c}e^{-\tau_{c}}\text{Ei}\left(-\tau +\tau_{c}\right)\right]+C_{m}$$ (22)

in the mid-infrared band, where $C_{f}$ and $C_{m}$ are integration constants. Over the surface temperatures considered here, transmission due to line absorption by water vapor is near one in the center of the water vapor window and near zero at the peak of the far- and mid-infrared bands. Thus, considering only line absorption by water vapor, $\int \tau e^{-\tau }d\nu_{j}=l_{j}$; including continuum absorption changes this result to $\int \tau e^{-\tau }d\nu_{j}=l_{j}e^{-\tau_{c}}\left[1-\text{Ei}\left(-\tau_{\text{min}}+\tau_{c}\right)\tau_{c}\right]$. These results, combined with $\int \tau e^{-\tau }d\nu_{c,j}=0$ (neglecting CO_2_), yield Eqs. 7 and 8, respectively. When including masking by CO_2_, Eq. 9 arises by differencing Eq. 21 across the CO_2_ band.

The derivation of $\frac{dH_{\text{sw}}}{{\mathrm{dT}}_{s}}$, the sensitivity of column-integrated shortwave heating to surface temperature, from Eq. 6 follows similar arguments. Integrating over all wave numbers in the near-infrared, we extract $I_{\nu }$ from the spectral integral and evaluate it at $\nu_{1,n}$, the wave number in the near-infrared band where τ=1 (*24*). This yields

$$\frac{dH_{\text{sw}}}{{\mathrm{dT}}_{s}}=\int_{0}^{\infty }\frac{dH_{\nu ,\text{sw}}}{{\mathrm{dT}}_{s}}d\nu =\frac{d\text{ln}\mathrm{WVP}}{{\mathrm{dT}}_{s}}I_{\nu }\left(\nu_{1,n}\right)\int \tau e^{-\tau }d\nu_{n}$$ (23)

where $\int d\nu_{n}$ is the integral over all wave numbers in the near-infrared band. Using the parameterization of water vapor spectroscopy in the near-infrared, the antiderivative of $\tau_{\nu ,\text{wv}}e^{-\tau_{\nu ,\text{wv}}}$ in the near-infrared is simply the transmission of water vapor multiplied by $l_{n}$. Because transmission is near one on the high wave number side of the near-infrared band and near zero on the low wave number side of the near-infrared band, Eq. 23 can be analytically integrated to obtain our broadband model for $\frac{dH_{\text{sw}}}{{\mathrm{dT}}_{s}}$, Eq. 11.
