# Supplementary material for: A spectroscopic theory for how mean rainfall changes with surface temperature
Source: Sci Adv. 2025 May 9;11(19):eadv6191. doi: 10.1126/sciadv.adv6191 (PMC12063646; doi:10.1126/sciadv.adv6191)
Supplement: Supplementary file 1 — Supplementary Text Figs. S1 to S5 Table S1 [file sciadv.adv6191_sm.pdf]

Science Advances
AAAS

Supplementary Materials for

A spectroscopic theory for how mean rainfall changes with surface temperature

Sean Cohen and Robert Pincus

Corresponding author: Sean Cohen, sean.cohen@columbia.edu

Sci. Adv. **11**, eadv6191 (2025)
DOI: 10.1126/sciadv.adv6191

This PDF file includes:

Supplementary Text
Figs. S1 to S5
Table S1

Supplementary Text

Surface Exchanges

In developing analytical expressions for the sensitivity of column-integrated longwave cooling to changes in surface temperature, we assumed that exchanges between the surface and atmosphere are small enough to be neglected. Here, we assess the validity of that assumption and outline the radiative mechanisms which act to keep surface exchanges small.

At each wavenumber ν, the contribution to column-integrated atmospheric cooling from radiative exchanges with the surface $Q_{\nu,\text{SX}}$ is given by

$$Q_{\nu,\text{SX}} = -\int_0^{\tau_\nu} \pi(B_\nu(T_s) - B_\nu(T(\tau'_\nu)))e^{-(\tau_\nu - \tau'_\nu)} d\tau'_\nu \tag{S1}$$

where τ_ν is the column optical depth, T_s is the surface temperature, and B_ν is the Planck emission function. Taking the derivative of this expression with respect to surface temperature, we obtain the sensitivity of surface exchanges to changes in surface temperature

$$\frac{dQ_{\nu,\text{SX}}}{dT_s} = -\frac{d}{dT_s}\left(\int_0^{\tau_\nu} \pi(B_\nu(T_s) - B_\nu(T(\tau'_\nu)))e^{-(\tau_\nu - \tau'_\nu)} d\tau'_\nu\right) \tag{S2}$$

which, using Leibniz's rule, we can simplify as

$$\frac{dQ_{\nu,\text{SX}}}{dT_s} = -\pi(B_\nu(T_s) - B_\nu(T(\tau_\nu)))\frac{d\tau_\nu}{dT_s} - \int_0^{\tau_\nu} \frac{d}{dT_s}\left(\pi(B_\nu(T_s) - B_\nu(T(\tau'_\nu)))e^{-(\tau_\nu - \tau'_\nu)}\right) d\tau'_\nu \tag{S3}$$

The first term vanishes under the assumption that the surface air temperature is equal to the surface temperature, while the second may be simplified under the assumption that atmospheric temperature at a given optical depth does not change with surface temperature ($\frac{dB_\nu(T(\tau_\nu))}{dT_s} = 0$) (*14, 15*). This yields

$$\frac{dQ_{\nu,\text{SX}}}{dT_s} = -\int_0^{\tau_\nu} \pi\frac{dB_\nu(T_s)}{dT_s}e^{-(\tau_\nu - \tau'_\nu)} d\tau'_\nu + \frac{d\tau_\nu}{dT_s}\int_0^{\tau_\nu} \pi(B_\nu(T_s) - B_\nu(T(\tau'_\nu)))e^{-(\tau_\nu - \tau'_\nu)} d\tau'_\nu \tag{S4}$$

$$= -\pi\frac{dB_\nu(T_s)}{dT_s}(1 - e^{-\tau_\nu}) - \frac{d\tau_\nu}{dT_s}Q_{\nu,\text{SX}} \tag{S5}$$

The second line is obtained by evaluating the first integral and using equation S1 to substitute for the second integral. The first term accounts for decreases in column-integrated cooling due to

increased surface emission ($\frac{dB_\nu(T_s)}{dT_s}$), while the second represents increases in column-integrated cooling due to increased moisture ($\frac{d\tau_\nu}{dT_s}$) which reduces the transmission between the atmosphere and the surface and thus damps the existing heating due to surface exchanges.

We approximate $Q_{\nu,\mathrm{SX}}$ by linearizing the Planck function in optical depth about the surface, that is, by letting $B_\nu(\tau'_\nu) = B_\nu(T_s) - \frac{dB_\nu(T_s)}{d\tau_\nu}(\tau_\nu - \tau'_\nu)$. Heating due to surface exchanges is dominated by the lowest levels of the atmosphere, where such a linearization is most accurate. Applying this linearization to Eq. S1 yields

$$Q_{\nu,\mathrm{SX}} = -\int_0^{\tau_\nu} \pi \frac{dB_\nu(T_s)}{d\tau_\nu}(\tau_\nu - \tau'_\nu)e^{-(\tau_\nu - \tau'_\nu)}d\tau'_\nu \tag{S6}$$

$$= -\pi \frac{\frac{dB_\nu(T_s)}{dT_s}}{\frac{d\tau_\nu}{dT_s}} \int_0^{\tau_\nu} (\tau_\nu - \tau'_\nu)e^{-(\tau_\nu - \tau'_\nu)}d\tau'_\nu \tag{S7}$$

$$= -\frac{\pi}{\alpha}\frac{dB_\nu(T_s)}{dT_s}\left(e^{-\tau_\nu} - \frac{1 - e^{-\tau_\nu}}{\tau_\nu}\right) \tag{S8}$$

where α is the relative rate of change of water vapor path, and thus optical depth ($\alpha = \frac{d\ln\tau_\nu}{dT_s}$), with surface temperature. This requires again neglecting any pressure dependence of absorption and assuming that most wavenumbers are dominated by emission by water vapor. To find the sensitivity of cooling due to surface exchanges, we take the derivative with respect to surface temperature:

$$\frac{dQ_{\nu,\mathrm{SX}}}{dT_s} = \pi \frac{dB_\nu(T_s)}{dT_s}\left(\frac{1 - e^{-\tau_\nu}}{\tau_\nu} - e^{-\tau_\nu}(1 + \tau_\nu)\right) \tag{S9}$$

where we have neglected terms including $\frac{d^2B_\nu(T_s)}{dT_s^2}$, since $\frac{d\ln\frac{dB_\nu(T_s)}{dT_s}}{d\ln T_s} \approx 2 - 4$ in the water vapor window, making $\frac{d^2B_\nu(T_s)}{dT_s^2} << \alpha\frac{dB_\nu(T_s)}{dT_s}$ at Earth-like surface temperatures.

Eq. S9 has several important implications. First, the sensitivity of cooling due to surface exchanges goes to zero for both very small and very large optical depth, indicating that spectral regions which are nearly completely opaque or transparent do not substantially contribute to $\frac{dQ_{\nu,\mathrm{SX}}}{dT_s}$. Second, the sensitivity of cooling due to surface exchanges is negative for $\tau_\nu < 1.8$, where increased surface emission reduces the sensitivity of integrated cooling to surface temperature, and positive for $\tau_\nu > 1.8$, where increased moisture increases the sensitivity. Water vapor optical depth spans many orders of magnitude in the atmosphere, suggesting that these mechanisms will largely cancel in the spectral integral.

Using Eq. S9, we can express the sensitivity of cooling due to surface exchanges as

$$\frac{dQ_{\mathrm{SX}}}{dT_s} = \int_0^\infty \frac{dQ_{\nu,\mathrm{SX}}}{dT_s} d\nu \approx \sum_j^2 \pi \frac{dB_\nu((\nu_{1.8,j}, T_s)}{dT_s} \int \left(\frac{1 - e^{-\tau_\nu}}{\tau_\nu} - e^{-\tau_\nu}(1 + \tau_\nu) \right) d\nu_j \qquad \text{(S10)}$$

where $\int d\nu_j$ is the integral over all wavenumbers in band j and $\nu_{1.8,j}$ is the wavenumber in band j where $\tau_{\nu,\mathrm{wv}} = 1.8$. Given that $\frac{dB_\nu(T_s)}{dT_s}$ varies slowly in wavenumber over the water vapor window, we neglect variation in the Planck function with wavenumber and use $\nu_{1.8,j}$ to represent emission in both the $\tau_\nu < 1.8$ and $\tau_\nu > 1.8$ spectral regions. Under line absorption alone, Eq. S10 integrates to zero over each absorption band because optical depth varies exponentially in wavenumber and because transmission due to line absorption by water vapor is near one in the center of the water vapor window and near zero at the peak of the far- and mid-infrared bands. When including continuum absorption, the sensitivity of cooling due to surface exchanges is larger, but nonetheless small in comparison to the sensitivity of cooling to space in the spectral integral (<10%, save at high surface temperatures when the sensitivity of cooling to space goes to zero).

Emission by Carbon Dioxide

Equation 4 in the main text was developed by assuming that non-condensable gases like carbon dioxide act only to mask changes in transmission due to water vapor. This assumption fails at surface temperatures exceeding about 310 K, as seen in the disagreement between line-by-line calculations and the analytical model. Here, we explain why this disagreement arises and how the analytical model can be adjusted accordingly.

Wavenumbers dominated by non-condensable emitters at a given concentration emit from a roughly constant pressure level. As the atmosphere warms, the temperature at a given pressure level in the troposphere increases, increasing emission from that wavenumber (*25, 30*). At most Earth-like surface temperatures, the majority of the CO_2 band emits from the stratosphere, where temperature profiles do not depend on surface temperature, so the primary impact of CO_2 is masking. However, the tropopause also rises with increasing surface temperature, which increases the range of wavenumbers dominated by carbon dioxide that emit from the troposphere. At very high surface temperatures (about 320 K), the tropopause is high enough that nearly the entirety

of the CO_2 band emits from the troposphere, violating the assumption that increased temperatures don't impact emission in the CO_2 band.

The expressions in the main text can be extended to account for increased emission from carbon dioxide under surface warming using a simplified version of the CO_2 "ditch" model presented in Ref (*25*). We assume that surface warming increases emission by carbon dioxide primarily by lifting the tropopause (*25*) and thus neglect changes in the total width of the CO_2 band and in emission at the edges of the CO_2 band with warming. This assumption is most valid at surface temperatures between about 290 K and 320 K, where the troposphere is warm enough that water vapor entirely masks surface emission at both edges of the CO_2 band but also cool enough that the entirety of the CO_2 band does not emit from the troposphere.

If the tropospheric lapse rate is vertically constant and emission by carbon dioxide is well-approximated by emission to space from its $\tau = 1$ temperature level (*25, 26*), then the relationship between emission temperature and wavenumber in the CO_2 band is linear. This allows us to express the relative change in emission from carbon dioxide with surface temperature as

$$\frac{d \ln Q_{\mathrm{CO_2}}}{dT_s} = \frac{d \ln \Delta\nu_{\mathrm{tropo}}}{dT_s} \tag{S11}$$

where $\Delta\nu_{\mathrm{tropo}}$ is the set of wavenumbers in the CO_2 band emitting from the troposphere. fig. S1 illustrates schematically how the spectral widening of tropospheric emission from carbon dioxide increases as the tropopause rises under warming.

To evaluate the right-hand side of Eq. S11, we require an approximation for $\Delta\nu_{\mathrm{tropo}}$. Assuming the outer edges of the CO_2 band are well-approximated by the wavenumbers for which the optical depth of carbon dioxide is unity at the surface, we can express $\Delta\nu_{\mathrm{tropo}}$ as

$$\Delta\nu_{\mathrm{tropo}} = 4 l_0 \ln\left(\frac{p_s}{p_t}\right) \tag{S12}$$

where p_s is the surface pressure, p_t is the tropopause pressure, and l_0 is the spectral width over which the absorption coefficient of carbon dioxide decays by a factor of e away from its peak. This allows us to rewrite Eq. S11 as

$$\frac{d\ln Q_{\mathrm{CO_2}}}{dT_s} = \frac{\frac{dp_t}{dT_s}}{p_t \ln\left(\frac{p_s}{p_t}\right)} \tag{S13}$$

Over the surface temperatures considered in this study, the right hand side of Eq. S13 varies from about 0.04 K^{-1} to 0.08 K^{-1}. Approximating Eq. S13 as a constant representative value $\frac{d\ln Q_{\mathrm{CO_2}}}{dT_s} \approx \gamma = 0.06\ \mathrm{K}^{-1}$, the absolute change in emission from carbon dioxide with surface temperature is

$$\frac{dQ_{\mathrm{CO_2}}}{dT_s} = \left(\frac{dQ_{\mathrm{CO_2}}}{dT_s}\right)_{T_s=T_0} e^{\gamma(T_s-T_0)} \tag{S14}$$

where T_0 is a reference surface temperature of our choice. When $T_s = 320$ K, the water vapor window closes entirely such that the sensitivity of column-integrated longwave cooling to surface temperature is largely unaffected by water vapor and thus is dominated by carbon dioxide. Hence, if we let $T_0 = 320$ K, we can set $\left(\frac{dQ_{\mathrm{CO_2}}}{dT_s}\right)_{T_s=T_0}$ equal to the sensitivity of column-integrated longwave cooling at 320 K, about 1 $\mathrm{W/m^2/K}$, as computed by line-by-line calculations. Increased cooling by carbon dioxide with surface temperature can therefore be expressed as:

$$\frac{dQ_{\mathrm{CO_2}}}{dT_s} = \left(1\mathrm{W/m^2/K}\right) e^{\gamma(T_s-320\mathrm{K})} \tag{S15}$$

In fig. S2, we demonstrate the impact of including these physics. While emission by carbon dioxide does not substantially alter the sensitivity of column-integrated longwave cooling at cooler surface temperatures, Eq. S15 notably improves our estimate of $\frac{dQ}{dT_s}$ at the warmest surface temperatures. This result is not predictive (since Eq. S15 is fit to line-by-line results) but rather illustrates the first order magnitude and temperature dependence of $\frac{dQ_{\mathrm{CO_2}}}{dT_s}$ as well as the mechanisms that control it. To the extent that a rising tropopause is the primary mechanism through which surface warming increases emission by carbon dioxide, $\frac{dQ_{\mathrm{CO_2}}}{dT_s}$ grows roughly exponentially. Once the entirety of the CO_2 band emits from the troposphere, however, this relationship breaks down.

Effect of Relative Humidity

Atmospheric cooling by spectrally-variable water vapor lines alone is nearly insensitive to relative humidity because the cooling profile, and hence the sensitivity of integrated cooling to surface

temperature, is dominated by the compensation between the near-exponential increase in water vapor path and the exponential decrease in absorption strength with wavenumber (*24*). Emission by the spectrally-gray water vapor continuum, on the other hand, depends strongly on the relative humidity. Cooling sensitivity peaks when the optical depth of the continuum (the product of water vapor path and absorption coefficient) is near unity; when column relative humidity is higher, sensitivity peaks at a cooler surface temperature. Figure S3 illustrates how peak sensitivity shifts to a lower surface temperature when relative humidity is increased from 70% to 100%. The magnitude of the shift is smaller in our analytical model than in line-by-line calculations because our idealized spectroscopy neglects the self-broadening of the continuum, which amplifies the shift.

Direct Response From Change in CO_2 Concentration

Increases in atmospheric CO_2 concentration directly impact the radiative energy budget of the atmosphere, even while surface temperature is held constant. Assuming the atmosphere equilibrates much more quickly than the surface does, this "direct response" can be a suitable proxy for the fast response, that is, the immediate change in global mean rainfall due to a change in CO_2 concentration before the surface temperature has had time to respond.

The direct response can be approximated by making the cooling-to-space approximation (*21*) and considering how much additional tropospheric emission the CO_2 band masks when its concentration changes. Since CO_2's absorption lines fall off roughly exponentially with wavenumber from the band center, ν_0, the additional masking generated by each side of the CO_2 band is proportional to the difference in the logarithm of the final (q_f) and starting (q_i) CO_2 concentrations, the spectral width over which the absorption coefficient of CO_2 decays by a factor of e away from its peak (l_0), and, of course, the tropospheric emission being masked ($\mathcal{F}_\nu$) (*26*). Assuming the CO_2 band is symmetrical and that variations in tropospheric emission are minimal across the CO_2 band, this yields:

$$\Delta Q_{\text{direct}} = -2\ln(\frac{q_f}{q_i})l_0\mathcal{F}_\nu \tag{S16}$$

where ΔQ_{direct} is the change in column-integrated tropospheric cooling due to a change in CO_2 concentration. Making the emission level approximation and assuming water vapor is the dominant

tropospheric emitter, we can write $\mathcal{F}_\nu$ as:

$$\mathcal{F}_\nu = \pi B_\nu(\nu_0, T_{\text{em}}) - \pi B_\nu(\nu_0, T_s)e^{-\tau} \quad \text{(S17)}$$

where T_s is the surface temperature, τ is the column optical depth due to water vapor at ν_0, and T_{em} is the "masked" emission temperature at ν_0, that is, the emission temperature that would be observed at ν_0 in the absence of CO_2. If water vapor's column optical depth is greater than 0.6, T_{em} is the temperature level at which water vapor's optical depth is equal to 0.6; otherwise T_{em} is equal to the surface temperature (*26*). The first term in Eq. S17 approximates the outgoing longwave radiation at the tropopause (*26*); the latter term approximates the portion of this outgoing longwave radiation which is coming from the surface rather than the troposphere. Combining Eq. S17 and Eq. S16 yields Eq. 12 in the main text, our approximation for the direct response due to a change in CO_2 concentration:

$$\Delta Q_{\text{direct}} = -2\ln(\frac{q_f}{q_i}) l_0 \pi \left(B_\nu(\nu_0, T_{\text{em}}) - B_\nu(\nu_0, T_s)e^{-\tau}\right) \quad \text{(S18)}$$

To validate Eq. S18, we compare it to full-physics line-by-line calculations in ARTS (fig. S4). As in the main text, we assume that tropospheric relative humidity is vertically constant (70%), that the tropospheric temperature follows a moist adiabat from the surface to a fixed temperature at the tropopause (220 K), that stratospheric humidity falls off vertically in accordance with the same moist adiabat, and that stratospheric temperature is held vertically constant at its value at the tropopause (220 K). Using the climate sensitivity derived from Ref. (*41*), we vary surface temperature and CO_2 concentration in tandem and assess the change in the radiative flux across the troposphere before and after a doubling of CO_2. Eq. S18 largely captures the first order magnitude of the LBL-diagnosed direct response over a wide range of surface temperatures. However, since we hold the stratospheric temperature constant, this validation neglects how the tropospheric energy budget is modified by changes in the stratospheric temperature profile, another facet of the fast response.

Interestingly, Eq. S18 implies that the direct response to a doubling of CO_2 – and by extension the fast response – does not depend directly on the concentration of CO_2 but only on surface

temperature and relative humidity. This result emerges from our idealization of carbon dioxide's spectroscopy; insofar as the CO_2 band is symmetrical and optically thick enough that its peak emits from the stratosphere, the direct response to a doubling of CO_2 depends only on the underlying emission by water vapor being masked. When surface temperature and CO_2 concentration are co-varied in accordance with a GCM-derived climate sensitivity (as in fig. S4), these idealizations prove reasonably strong. However, at low CO_2 concentration and high surface temperature, the center of the CO_2 band emits from the troposphere, causing the masking by CO_2 to be much smaller than Eq. S18 would predict. Similarly, at high CO_2 concentration and low surface temperature, CO_2's 1000 cm^{-1} band dominates tropospheric emission in the water vapor window such that increases in carbon dioxide actually increase tropospheric cooling, causing the direct response to reverse sign.

Hydrological Sensitivity in a Grey, Optically-thin Atmosphere

The fact that column-integrated water vapor grows with surface temperature notably more quickly than mean rainfall does (*4*) seems to suggest that hydrological sensitivity is untethered from thermodynamic constraints. However, since water vapor is Earth's main greenhouse gas, the rate at which it increases with surface warming is closely tied to the sensitivity of column-integrated radiative cooling with surface temperature and, insofar as the radiative constraint on mean rainfall holds, to hydrological sensitivity as well. Mean rainfall's comparatively sluggish growth with surface temperature emerges from the additional constraints imposed by water vapor's spectroscopy.

The role of spectroscopy in shaping hydrological sensitivity is highlighted with a thought experiment: what if water vapor's spectroscopy were gray and optically-thin? Neglecting shortwave heating and all emitters other than water vapor, the sensitivity of column-integrated radiative cooling to surface temperature would be

$$\frac{dQ_{\nu,\text{thin}}}{dT_s} = -\pi B_\nu(T_s)\frac{d\mathcal{T}}{dT_s} = \pi B_\nu(T_s)\frac{d\tau}{dT_s}\mathcal{T} \approx \pi B_\nu(T_s)k\frac{dWVP}{dT_s} \quad \text{(S19)}$$

where $\tau_\nu = \tau$ (grey), $\mathcal{T} \approx 1$ (optically thin), and $\frac{d\tau}{dT_s} = k\frac{dWVP}{dT_s}$ (water vapor atmosphere). Here, k is water vapor's grey mass absorption coefficient and WVP is the column-integrated water vapor. Integrating over all wavenumbers:

$$\frac{dQ_{\text{thin}}}{dT_s} = k\frac{dWVP}{dT_s}\int_0^{\infty}\pi B_\nu(T_s)d\nu = k\alpha WVP\sigma T_s^4 \tag{S20}$$

where we let $\frac{dWVP}{dT_s} = \alpha WVP$, with α is the constant logarithmic sensitivity of water vapor path to surface temperature. Here, k and α are constants, so the surface temperature dependence of column-integrated cooling is set entirely by the product of water vapor path and Planck emission. From a typical tropopause temperature (220 K) to a typical surface temperature (300K), Planck emission grows by a factor of about 3, while water vapor path grows by several orders of magnitude. Hence, water vapor path dominates the temperature dependence of Eq. S20, causing column-integrated cooling to grow roughly with Clausius-Clapeyron (slightly faster, in fact, due to the additional impact of Planck emission).

Effect of Surface Sensible Heating

One way to adjust the radiative constraint on mean rainfall to account for surface sensible heat fluxes is to use the free-atmospheric (rather than the total-atmospheric) radiative cooling as a proxy for mean rainfall (*7, 8*). We claim in the main text that our analytical models for total-atmospheric radiative cooling sensitivity ($\frac{dQ}{dT_s}$) can be adjusted to approximate the sensitivity of free-atmospheric radiative cooling to surface temperature ($\frac{dQ_{\text{free}}}{dT_s}$) by simply replacing the surface temperature T_s with the temperature at the bottom of the free troposphere T_f. This yields Eq. 12 in the main text. Conceptually, Eq. 12 is rooted in the ideas presented in Ref. (*8*): The troposphere's flux divergence profile is largely independent of surface temperature, so the sensitivity of free-atmospheric radiative cooling to surface temperature primarily depends on the flux divergence at the bottom of the free troposphere. We assess the validity of Eq. 12 in fig. S5 by comparing our adjusted analytical model to line-by-line calculations. Following Ref. (*7*), we assume the 860 hPa pressure level to be the bottom of the free troposphere. Our adjusted analytical model reasonably captures how free-atmospheric net cooling changes with surface temperature (save at high surface temperatures, where emission by carbon dioxide dominates; see section above on *Emission by Carbon Dioxide*).

To more formally derive Eq. 12, we consider the upward flux at the bottom of the free atmosphere, $\mathcal{F}_\nu$. Assuming the temperature level T_f has optical depth τ_ν^* (with column optical depth τ_ν) at wavenumber ν, we have:

$$\mathcal{F}_\nu = \pi B_\nu(T_s)e^{-(\tau_\nu-\tau_\nu^*)} + \int_{\tau_\nu^*}^{\tau_\nu} \pi B_\nu(\tau_\nu')e^{-(\tau_\nu'-\tau_\nu^*)}d\tau_\nu' \tag{S21}$$

Since $\frac{d^2B_\nu}{d\tau_\nu^2} \approx 0$ in the lower troposphere, we can linearize the blackbody emission between τ_ν and τ_ν^*:

$$B_\nu(\tau_\nu') = B_\nu(\tau_\nu) + \frac{B_\nu(T_s) - B_\nu(\tau_\nu^*)}{\tau_\nu - \tau_\nu^*}(\tau_\nu' - \tau_\nu^*) \tag{S22}$$

This yields

$$\mathcal{F}_\nu = \pi B_\nu(\tau_\nu^*) + \pi(B_\nu(T_s) - B_\nu(\tau_\nu^*))\left(\frac{1-e^{-(\tau_\nu-\tau_\nu^*)}}{(\tau_\nu-\tau_\nu^*)}\right) \tag{S23}$$

which we can rewrite as

$$\mathcal{F}_\nu = \pi B_\nu(\tau_\nu^*)\left(1 + \frac{(B_\nu(T_s) - B_\nu(\tau_\nu^*))}{B_\nu(\tau_\nu^*)}\left(\frac{1-e^{-x}}{x}\right)\right) \tag{S24}$$

where $x = \tau_\nu - \tau_\nu^*$. Letting $B_\nu(T_s) - B_\nu(\tau_\nu^*) = \frac{dB_\nu(\tau_\nu^*)}{dT(\tau_\nu^*)}(T_s - T(\tau_\nu^*))$ yields

$$\mathcal{F}_\nu = \pi B_\nu(\tau_\nu^*)\left(1 + \left(\frac{T_s}{T(\tau_\nu^*)} - 1\right)\frac{d\ln B_\nu(\tau_\nu^*)}{d\ln T(\tau_\nu^*)}\left(\frac{1-e^{-x}}{x}\right)\right) \tag{S25}$$

Since $\frac{T_s}{T(\tau_\nu^*)} - 1$ is at most 0.04, $\frac{d\ln B_\nu(\tau_\nu^*)}{d\ln T(\tau_\nu^*)}$ is at most 7 (between 150 and 1500 cm^{-1}), and $\frac{1-e^{-x}}{x}$ is at most 1, $\mathcal{F}_\nu$ is at most only about 25% larger than $\pi B_\nu(\tau_\nu^*)$.

The sensitivity of $\mathcal{F}_\nu$ to surface temperature has a similar magnitude deviations from $\pi\frac{dB_\nu(\tau_\nu^*)}{dT_s}$. Taking the derivative of $\mathcal{F}_\nu$ with respect to surface temperature and assuming $\frac{dB_\nu(T_s)}{dT_s} = \frac{dB_\nu(\tau_\nu^*)}{dT_s}$ (changes in lapse rate are minimal in the boundary layer) yields:

$$\frac{d\mathcal{F}_\nu}{dT_s} = \pi\frac{dB_\nu(\tau_\nu^*)}{dT_s} + \pi(B_\nu(T_s) - B_\nu(\tau_\nu^*))\left(\frac{xe^{-x} - 1 + e^{-x}}{x^2}\right)\frac{dx}{dT_s} \tag{S26}$$

Assuming that water vapor is the dominant emitter at most wavenumbers ($\frac{dx}{dT_s} = x\alpha$) and that temperature deviations are small in the boundary layer ($B_\nu(T_s) - B_\nu(\tau_\nu^*) = \frac{dB_\nu(\tau_\nu^*)}{dT_s}(T_s - T(\tau_\nu^*))$), we have:

$$\frac{d\mathcal{F}_\nu}{dT_s} = \pi \frac{dB_\nu(\tau_\nu^*)}{dT_s} \left(1 + \alpha(T_s - T(\tau_\nu^*)) \left(\frac{xe^{-x} - 1 + e^{-x}}{x}\right)\right) \tag{S27}$$

Since α is at about 0.085 K^{-1}, $(T_s - T(\tau_\nu^*))$ is at most 9 K, and $\frac{xe^{-x}-1+e^{-x}}{x}$ is at most 0.3, $\frac{d\mathcal{F}_\nu}{dT_s}$ is at most only 25% larger than $\pi\frac{dB_\nu(\tau_\nu^*)}{dT_s}$. Given that the upward flux at the bottom of the free atmosphere is approximately equal to the flux that would be produced by an imaginary surface at this temperature level ($\mathcal{F}_\nu \approx \pi B_\nu(\tau_\nu^*)$) and given that these quantities change with surface temperature at similar rates ($\frac{d\mathcal{F}_\nu}{dT_s} \approx \pi\frac{dB_\nu(\tau_\nu^*)}{dT_s}$), we can establish a mapping between the radiative transfer problem for the total atmosphere and the radiative transfer problem for the free atmosphere. This mapping allows us to use Eq. 12 to approximate the free-atmospheric radiative cooling sensitivity using our analytical models for total-atmospheric radiative cooling sensitivity.

Parameter Values

For reference, we list all of the thermodynamic and spectroscopic parameters used in our simple broadband and spectral models in Table S1. Note that what we call the ”far-infrared” is truly the long-wavelength infrared; we make this change in nomenclature for simplicity.

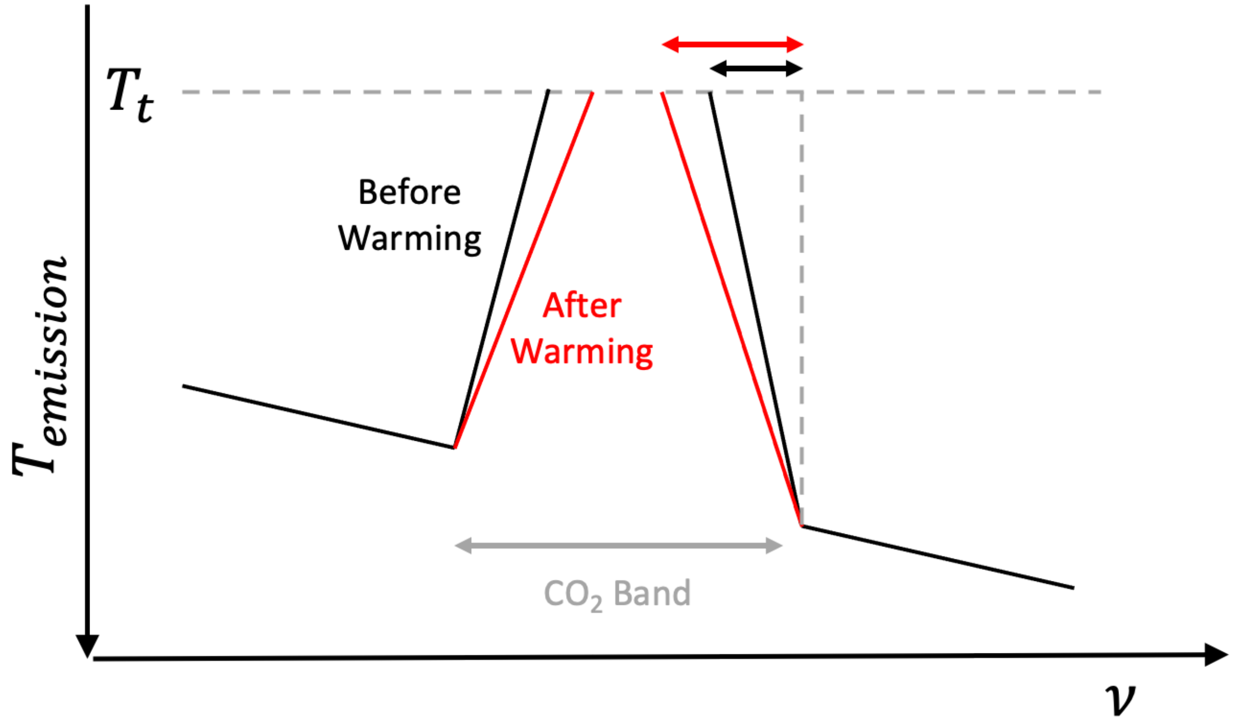

Figure S1: **Schematic of our simple model for increases in CO_2 emission with surface temperature.** Neglecting changes in the total width of the CO_2 band and in the emission temperature at the edges of the CO_2 band, the relative change in CO_2 emission with surface temperature (black to red emission temperature lines) is well-approximated by the relative change in the spectral width of portion of the CO_2 band emitting from the troposphere (black to red arrows). This approximation breaks down once the entirety of the CO_2 band emits from the troposphere (at surface temperatures in excess of about 320 K).

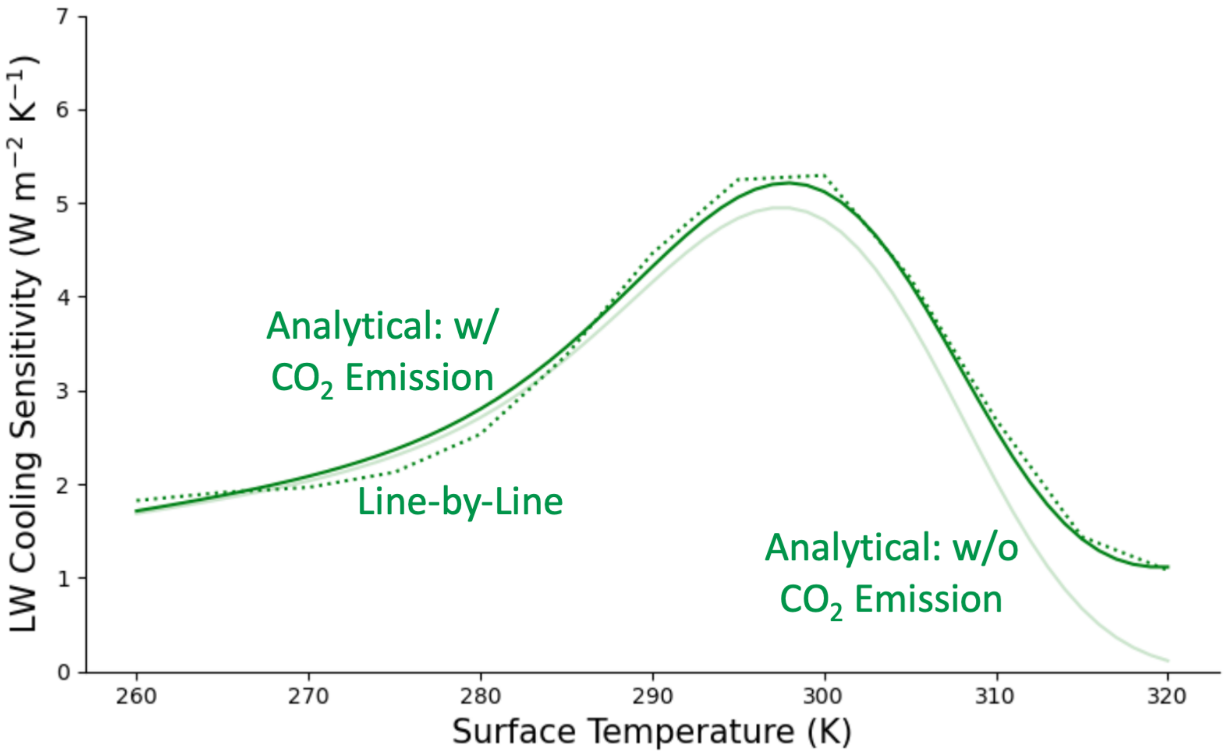

Figure S2: **Sensitivity of column-integrated longwave cooling to changes in surface temperature for an atmosphere of water vapor and carbon dioxide.** The faded line shows results from our broadband model (assuming carbon dioxide solely masks changes in emission by water vapor). The solid line shows results from our broadband model plus contributions to column-integrated longwave cooling sensitivity from emission by carbon dioxide (Eq. S15). The dotted line shows the sensitivity derived from the full-physics, line-by-line radiative transfer model ARTS.

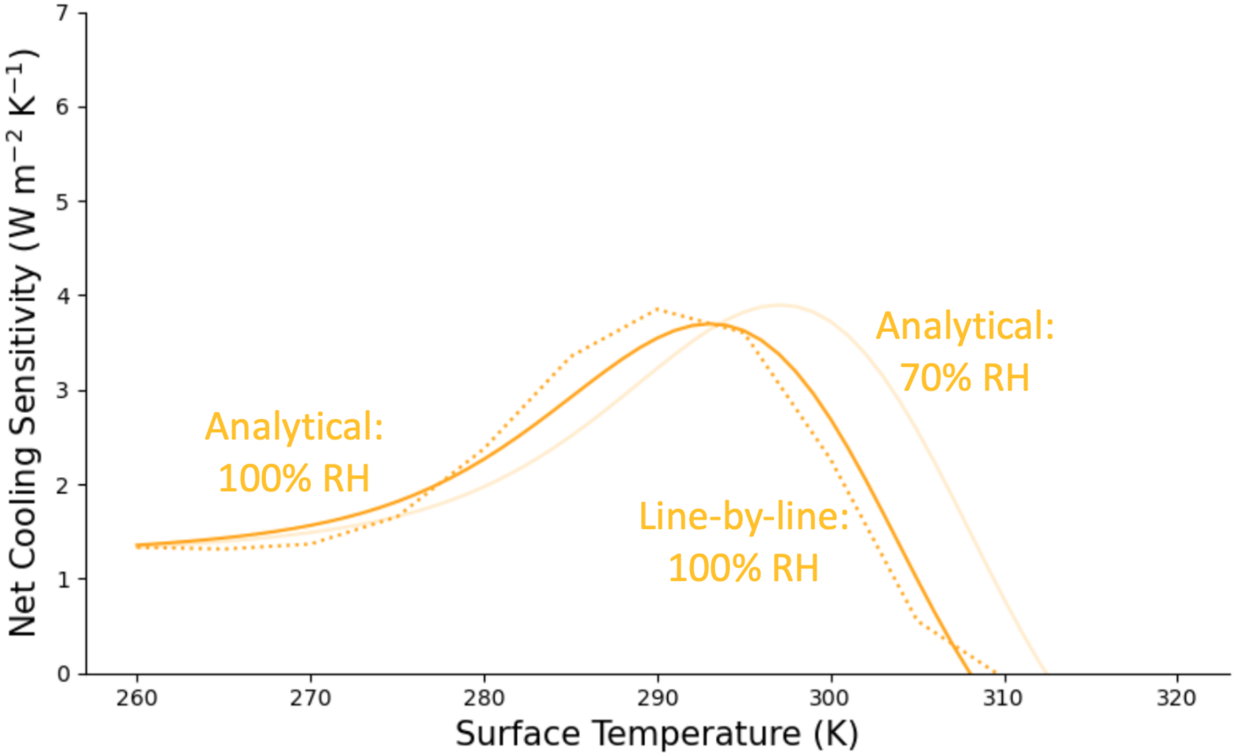

Figure S3: **Sensitivity of column-integrated net (longwave and shortwave) radiative cooling to changes in surface temperature for a saturated (100% RH) column.** The solid line shows results from our broadband model, the dotted line shows results from the full-physics, line-by-line radiative transfer model ARTS, and the faded line (for reference) shows results from our broadband model for a column with 70% RH. Our broadband model notably under-predicts the shift in peak sensitivity because we neglect the direct temperature dependence of the water vapor continuum.

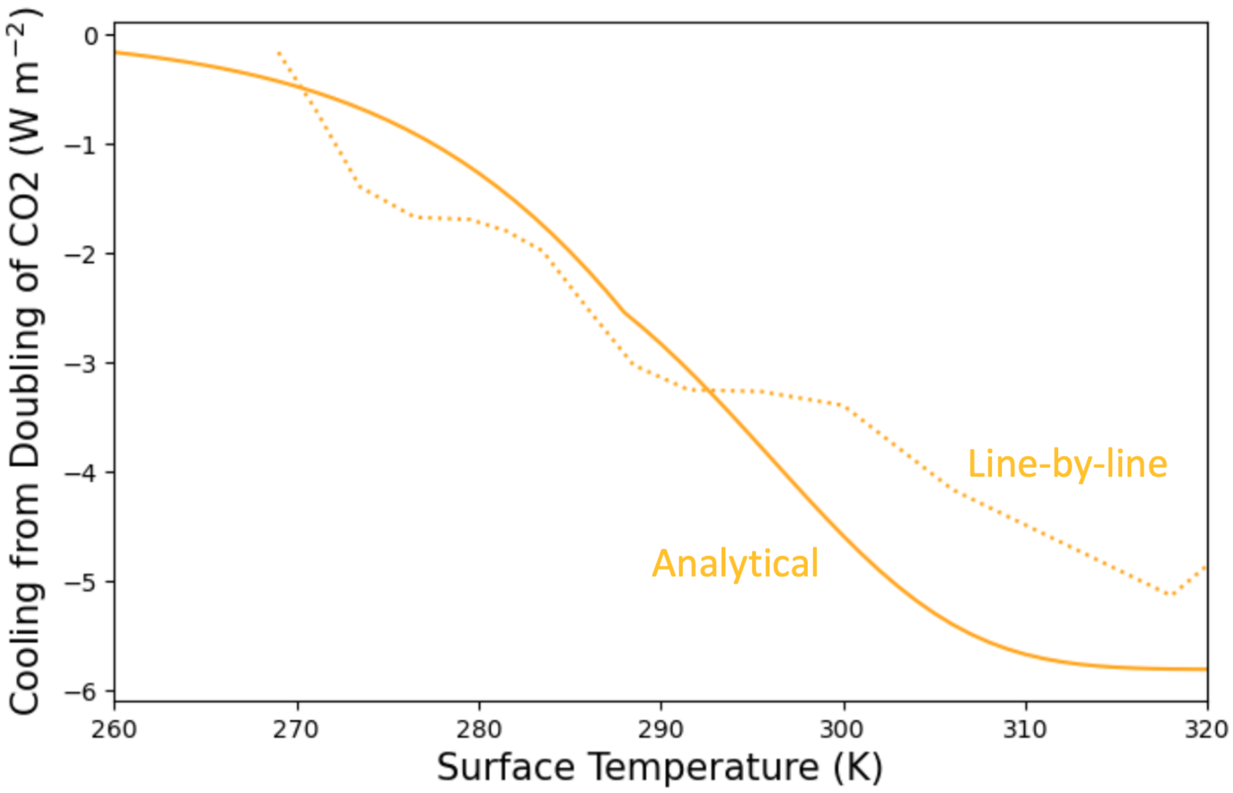

Figure S4: **Change in column-integrated tropospheric cooling due to a doubling of CO_2 (i.e., the direct response) for a column with 70% RH.** The solid line shows results from Eq. S18 and the dotted line shows results from the full-physics, line-by-line radiative transfer model ARTS. Masking by CO_2 is minimal at low surface temperatures and grows as the optical depth of water vapor in the CO_2 band increases with surface temperature.

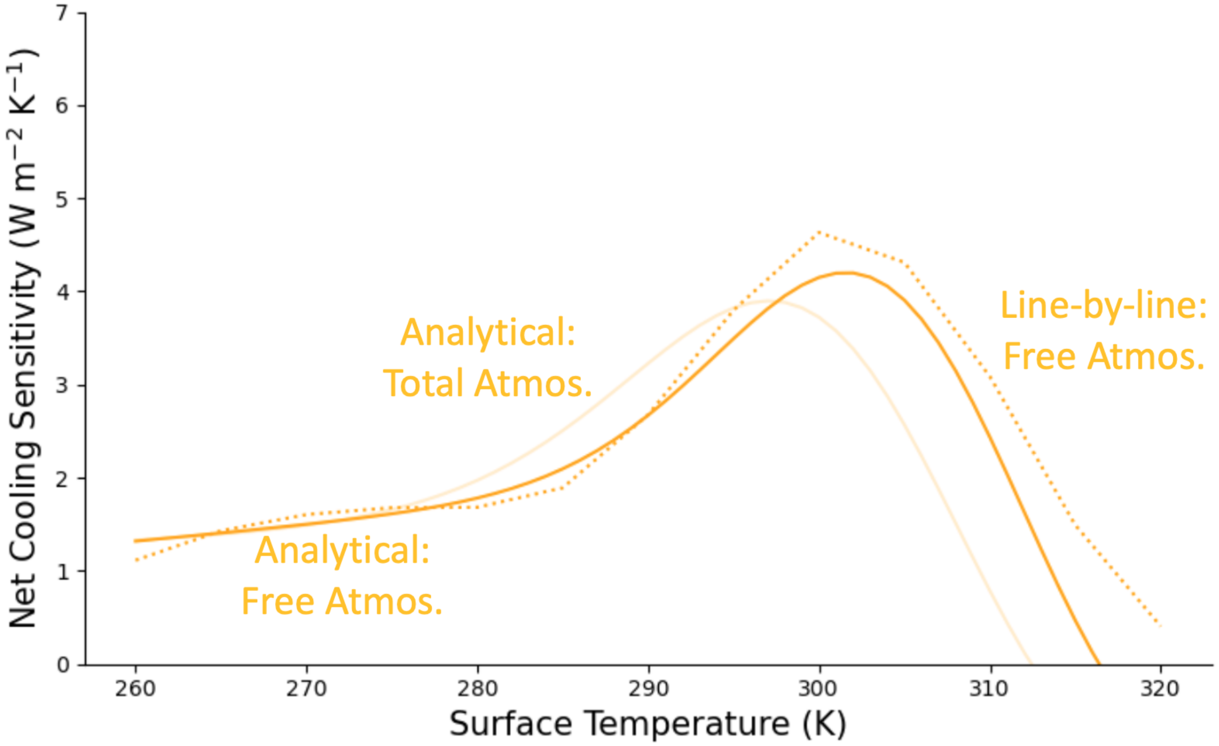

Figure S5: **Sensitivity of free-atmospheric net (longwave and shortwave) radiative cooling to changes in surface temperature.** The solid line shows results from our (adjusted) broadband model and the dotted line shows results from the full-physics, line-by-line radiative transfer model ARTS. For reference, the faded line shows the sensitivity of total-atmospheric net radiative cooling to changes in surface temperature.

Table S1: **List of parameters used in our simple spectral and broadband models**

Parameter	Description	Assumed Value
α	Relative rate of change of water vapor path with surface temperature	0.085 K^{-1}
D	Diffusivity factor	5/3
k_f	Absorption cross-section at peak of far-infrared band	165 m^2/kg
k_m	Absorption cross-section at peak of mid-infrared band	15 m^2/kg
k_n	Reference absorption cross-section of near-infrared band	7.1 m^2/kg
k_0	Absorption cross-section at center of CO_2 band	500 m^2/kg
k_{cont}	Absorption cross-section of water vapor continuum	0.011 m^2/kg
l_f	Decay rate of absorption cross-section in far-infrared band	55 cm^{-1}
l_m	Decay rate of absorption cross-section in mid-infrared band	38 cm^{-1}
l_n	Decay rate of absorption cross-section in near-infrared band	890 cm^{-1}
l_0	Decay rate of absorption cross-section in CO_2 band	10.2 cm^{-1}
ν_f	Wavenumber of the peak of the far-infrared band	150 cm^{-1}
ν_m	Wavenumber of the peak of the mid-infrared band	1500 cm^{-1}
ν_0	Wavenumber of the peak of the CO_2 band	667.5 cm^{-1}
